# Relationship between the education level and dermatological lesions in feet of diabetic patients

**DOI:** 10.1186/1758-5996-7-S1-A187

**Published:** 2015-11-11

**Authors:** Imaikon Gomes de Lima, Amanda Soares Peixoto, Alana Ferreira de Oliveira, José Nunes Borges, Joelma Lobo Flórence da Costa, Miguel Soares Pancieri, Geane Oliveira Silva, Carla Andréa Avelar Pires, Cézar Augusto Muniz Caldas

**Affiliations:** 1 Universidade Federal Do Pará, Ananindeua, Brazil

## Background

Diabetes mellitus is a heterogeneous group of metabolic disorders that result in hyperglycemia by defects in insulin secretion or action, or both. This disease has a direct association with long-term damage to the body, including vascular disease and peripheral neuropathy, increasing the risk of foot ulcers and amputations. There are several risk factors that contribute to the development of diabetic foot ulcers, such as the duration of the disease, uncontrolled metabolism and the mishandling of the foot. Effective health actions related to education about the disease and, more specifically, on the diabetic foot may avoid or delay diabetic foot ulcers.

## Objectives

To characterize the most common dermatological lesions on the feet of diabetic patients of endocrinology service in a university hospital and its relationship to the level of education.

## Materials and methods

A descriptive, retrospective study from data collected during the execution of the extension project in the period of 2014 to 2015. The Patients who agreed to participate in the project underwent a targeted physical examination seeking to identify and characterize dermatological lesions present in their feet.

## Results

Among the 175 covered diabetic patients, 125 (71.4%) were female. The average age was 57.2±12.4 yrs., with 60% of patients under 60 yrs. About the education level, 5.1% said they had never been to school and 47.4% did not conclude elementary school. The dermatological examination revealed the presence of skin lesions on the feet in 94.3% of patients addressed. Patients with lower education had higher number of lesions per person (average of 1.92 lesions) compared to patients with completed secondary education (average of 1.54 injuries). Among the dermatological changes that stand out are xerosis (55.4%), onychomycosis (46.9%), fissures (33.7%), and the calluses (14.3%). Only 5.7% had no lesions. The most frequent associations were xerosis and fissures. The amputation occurred in 5 (2.9%) patients.

## Conclusion

It can be concluded that is high the frequency of risk conditions for the development of ulcerations in the population assisted by the project and the low level of education can be a contributing factor to this situation.

